# Factors influencing the impact of ex-post legislative evaluations: a scoping review

**DOI:** 10.1080/13572334.2023.2268320

**Published:** 2023-10-25

**Authors:** Linda J. Knap, Roland D. Friele, Rob van Gameren, Johan Legemaate

**Affiliations:** a Netherlands Institute for Health Services Research (Nivel), Utrecht, The Netherlands; b Tranzo Scientific Center for Care and Wellbeing, Tilburg University, Tilburg, The Netherlands; c University of Amsterdam, Law Centre for Health & Life, Amsterdam, The Netherlands

**Keywords:** Impact, influence, ex-post legislative evaluation, post-legislative scrutiny, scoping review

## Abstract

This article explores the factors that influence the impact of ex-post legislative evaluations and suggests that these factors can be divided into three main categories: context, research quality, and interaction. Contextual factors, including the evaluation’s initiation, it’s place in the legislative process, the varied functions given by stakeholders, and the level of political or social attention, are beyond researchers’ control. However, researchers can influence research quality and interaction with stakeholders, such as the evaluations’ commissioner, as well as the society at large, thereby increasing the likelihood of achieving impactful results. They should engage with the evaluation context to improve impact, but must also maintain independence while being influenced by the context. These findings are in line with the much broader literature on the impact of policy and programme evaluations which pays less attention to the policy instrument legislation. Therefore, both disciplines have an interest in a better exchange of knowledge.

## Introduction

Ex-post legislative evaluations, also known as post-legislative scrutiny, offer insights into the practical functioning of legislation following its enactment. These evaluations are crucial within the legislative process, concentrating specifically on assessing the effectiveness of legislation, a government instrument with significant societal impacts. Despite legislation’s profound impact on society, such as in critical sectors like healthcare, previous research has indicated that the impact of ex-post legislative evaluations is largely confined to legislative and political domains, with limited impact on society at large (Knap et al., 2023). This is concerning, given the anticipated substantial benefits for the general public through effective evaluations (Moulds & Khoo, 2020). Various scholars have emphasised the need to comprehend the factors that influence or enhance the impact of ex-post legislative evaluations (Doust & Hastings, 2019; Van Voorst & Zwaan, 2019), creating a gap in the literature that underscores the importance of conducting a dedicated study on these influences. Such research will enrich our understanding of the conditions fostering successful evaluation and their ultimate impact on pertinent domains.

This scoping review exclusively focuses on ex-post legislative evaluation literature due to the unique characteristics and complexities of this practice, setting it apart from other types of policy evaluations. Combining empirical and legal research, legislative evaluations impact not only the political or policy sphere, but also the legal domain, shaping legislation design (Knap et al., 2023). Despite this common ground, there are significant differences emerge in the global conduct of these evaluations. Concentrating on this distinct policy evaluation type proves valuable, an approach already acknowledged in existing literature (for example: Knap et al., 2023; Van Voorst, 2018; Zwaan et al., 2016; Van Aeken, 2011). Ex-post legislative evaluations often hold a unique position within academic discourse, with minimal exploration in the broader context of policy and programme evaluation. Therefore, this scoping review aims to delve into the depth of knowledge and insights of the ex-post legislative evaluation domain concerning impact, ensuring the relevance of this study’s findings in this specialised field.

In the field of and policy and programme evaluations, a longstanding of research focuses on the factors that influence the utilisation of such evaluations. Since the late 1900s, an ongoing discourse has surrounded the utilisation of evaluations, with notable contributions of Alkin et al. (1979), Patton (1988), and Weiss (1979). Central to this discourse is the repeated exploration of the definition of ‘use’ and the broader concept of ‘influence’ (Alkin & King, 2017; Henry & Mark, 2003; Kirkhart, 2000; Leviton & Hughes, 1981). The broader evaluation literature has identified numerous factors that can foster the utilisation of evaluations. These include the relevance of the evaluations and the quality of their dissemination (timeliness, credibility, quality of presentations and means of dissemination, as well as incentives and capacities) (Feinstein, 2002). Additionally, the degree of polarisation and the distribution of costs between producers and users (Contandriopoulos & Brousselle, 2012), as well as the organisational context in which evaluations are conducted (Højlund, 2014) are important factors. To facilitate the utilisation of evaluations, various frameworks have been proposed. One such framework zeroes in on five clusters of variables that influence utilisation and posits hypotheses about the reasons for their effects (e.g. relevance, communication, information processing, credibility, user involvement and advocacy) (Leviton & Hughes, 1981). Another framework proposed a distinction between the utilisation of evaluations (e.g. the evaluative culture and organisational context and user characteristics) and the usability of evaluations (e.g. the evaluation design process and evaluation design quality) (Saunders, 2012).

In a more recent publication, Alkin and King (2017) highlight essential factors that affect evaluation usage. These factors were drawn from three significant studies: Cousins and Leithwood (1986), Shulha and Cousins (1997), Johnson et al. (2009), and the Program Evaluation Standards by Yarbrough et al. (2010).

The authors observed that these studies identified similar factors, resulting in a significant overlap between them. Alkin and King categorise these factors into four groups:

(1) User factors: Users’ positive prior experiences and meaningful involvement in the evaluation impact their predispositions towards evaluation. An explicit commitment to use evaluations is crucial within the ‘user factors’ category.
(2) Evaluator factors: The evaluator’s commitment to stimulate use and engage potential users, as well as their political sensitivity and credibility, are significant factors in the evaluation process. Establishing a good working relationship and involving users in the evaluation’s conduct can also impact the evaluator’s credibility.
(3) Evaluation factors: The third category pertains to the evaluation itself, including procedures, relevance of information, and communication quality. Appropriateness and credibility of methods are more important than technical excellence. Information must meet users’ needs, and communication should be understandable and timely.
(4) Organisational/social context factors: The nature of the organisation in which an evaluation is conducted has a substantial impact on the successful achievement of evaluation use. Various factors include organisational characteristics of the program, unit level autonomy, institutional arrangements, and external factors like the community and other agencies. Other sources of information beyond the evaluation are likely to be employed in decision-making.

It is worth noting that these four groups of factors are all part of the context of any evaluation, as per the authors’ observations.

These established notions about the utilisation of evaluations in general could contribute to the specific doctrine on the impact of legislative evaluations. In this scoping review, we aim to analyse the existing literature concerning the factors influencing the impact of ex-post legislative evaluations, both at the European and national legislative levels. We will approach this from the researchers’ perspective on the evaluation process. Subsequently, we will reflect on how these factors align with the broader evaluation literature’s discourse on evaluation utilisation. This approach aims to take an initial step toward integrating these spheres.

### Research method

This study aims to present the insights derived from literature on the factors that influence the impact of ex-post legislative evaluations in order to identify the available evidence on the issue, and analyse knowledge gaps in this field (Colquhoun et al., 2014). For this reason, this scoping review has followed the methodological PRISMA-ScR framework (Arksey & O’Malley, 2005; Levac et al., 2010). Scoping reviews are an ideal tool to determine the scope or coverage of a body of literature on any given topic that is not well charted yet (in this case, the factors that influence the impact of expost legislative evaluations) and provide an overview (broad or detailed) of the literature’s focus. This scoping review is based on the same dataset as our previous scoping review on the different types of impact of ex-post legislative evaluations (Knap et al., 2023). Despite the fact that the data was analysed with a different research question, the description of the first three phases of this method section contains similarities with the previous scoping review.

Additionally, these findings will be reflected according to the categorisation presented in the introduction of the article, which is derived from a broader evaluation literature classification (Alkin & King, 2017).

#### Phase 1

In an effort to capture all relevant literature, the study started with a broad research question: *What can be found in the scientific literature about the methodology and impact of ex-post legislative evaluations*? To ensure a broad search strategy, the research question did not include any specific jurisdiction or field of law. After conducting a detailed search, the research question was narrowed (see phase 4).

#### Phase 2

A search was conducted of the Web of Science, Worldcat and Legal Intelligence scientific databases using different search strings for an initial scope of the scientific literature (first quarter of 2021). Given that literature on this topic was expected to be scarce, no timespan was selected for the search.

Identical search strings were applied for each database with both English and Dutch search terms, using Boolean operators (AND, OR), a wildcard symbol, quotation marks, parenthesis and truncation in order to improve the search strategy. We initially started with a broad search strategy followed by two more specific search strategies, one related to methodology and the other to impact. Synonyms were applied for this purpose. The final search strings are included in Table 1.

#### Phase 3

**Figure 1:**
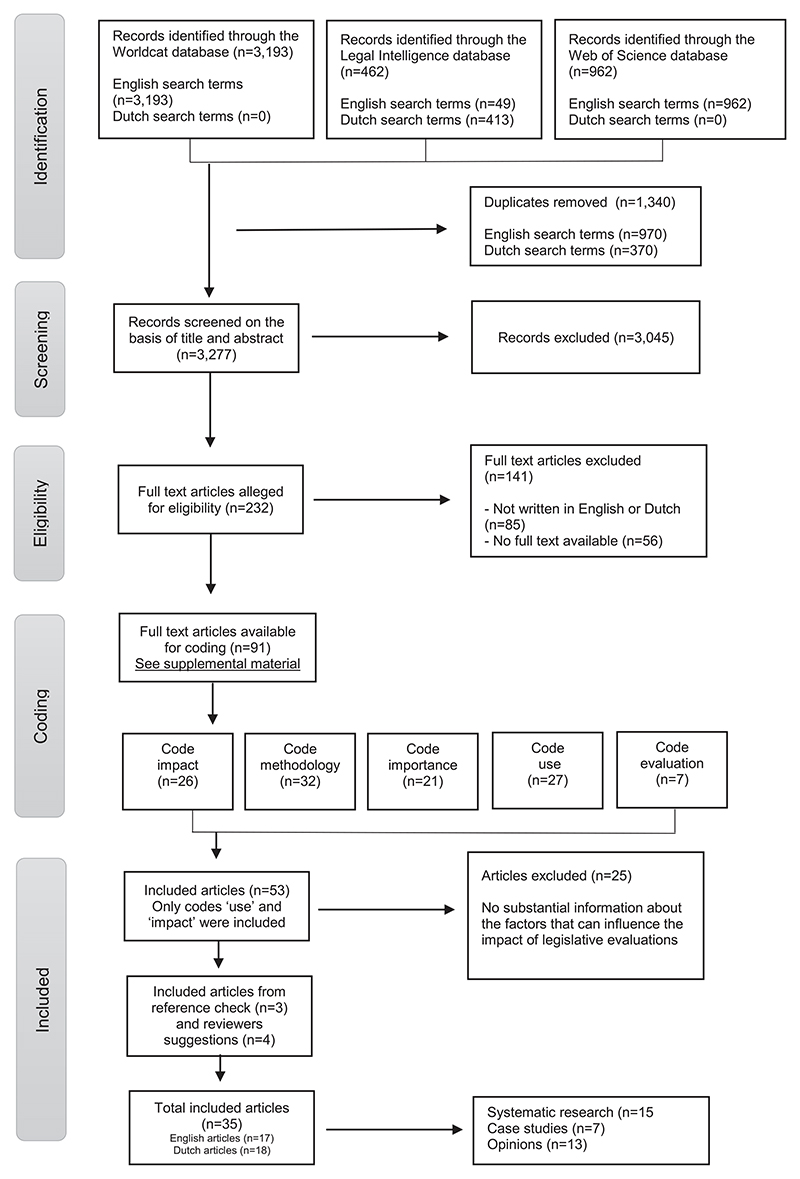
Methods flowchart.

In total, 4,204 studies were found with English search terms, and 413 studies were found with Dutch search terms (see Figure 1 for the entire search process, which is explained in more detail below). All literature was uploaded in Rayyan software, an administrative tool that facilitates the process of identifying and selecting studies when conducting a systematic literature review (Ouzzani et al., 2016). After merging the data, duplicates were removed (1,340 out of 4,617 studies). The literature was screened and selected by the first author (LK) based on title (for example, excluding titles that were not on the subject of ex-post legislative evaluations) and abstract. Independently, another author (RvG) reviewed a random selection of 5% (164 studies) using the initial criteria. For 7 of the 164 studies, disagreements between the authors about the selection had to be resolved through discussion.

Studies were deemed relevant if they fully or partly focused on the methodology and/or impact of ex-post legislative evaluations and were written in English or Dutch. Since the Netherlands has a long history of ex-post legislative evaluations and much literature is written in Dutch, the research group considered this a valuable addition to the English-language literature. During the selection of studies, those on the impact of legislation itself rather than the impact of ex-post legislative evaluations were excluded, as were studies on the impact of evaluations in general without a reference to expost legislative evaluations. Policy evaluations without a legal aspect were also excluded. Finally, ex-ante legislative evaluations were excluded from this study due to the different research designs used and their significance, as they are normally conducted before legislation is passed. On this basis, a total of 3,045 out of 3,277 studies were excluded and 232 studies were included (see Figure 1).

After selecting the studies and, in order not to miss any relevant coding, authors (LK) and (RvG) coded the studies based on title and abstract without agreeing on the codes beforehand. After, all authors agreed on the coding for the full text assessment; namely, methodology, use, impact, importance, and evaluation. Differences of opinion in the assigned types of coding after full text assessment were resolved through discussion between the authors. The full text versions of all 232 studies were manually searched. The studies that were either unavailable in full text (*n* = 56) or not written in English or Dutch (*n* = 85) were excluded (141 out of 232 studies). With regard to data openness, a list of the remaining 91 articles is included in the supplemental material.

#### Phase 4

In phase 4, the research question was narrowed down: *What can be found in the scientific literature about the factors that influence the impact of ex-post legislative evaluations?* As it appeared that much had already been written on the methodology of these evaluations, the focus on their impact could make a greater scientific contribution. For this purpose, a filtered selection was made of only studies coded with ‘impact’ (26 out of 91 studies) or ‘use’ (27 out of 91 studies). These 53 studies were fully read and assessed for relevance by both authors (LK) and (RvG), after which 28 studies were included (see Figure 1). References from the relevant studies were hand-searched by authors (LK) and (RvG) resulting in three additional studies that were added to the number of included studies. A final addition was made based on suggestions by reviewers. Due to the strict focus on expost legislative evaluations, the term ‘post-legislative scrutiny’ fell outside the scope of included data. Reviewers of the previous article pointed this out, so this term was manually searched in all three databases, after which four studies were added to the dataset. Based on the methods used, three types of studies were distinguished: systematic research in which a certain number of ex-post legislative evaluations were studied in a systematic manner (15 out of 35); case studies (7 out of 35); and expert opinions (13 out of 35). Importance was allocated to the included literature in this order and opinions could be empirically verified by case studies or systematic research. In the results of this scoping review, specific reference is made to these three types of studies.

## Results

The reviewed literature reveals several factors that influence the impact of expost legislative evaluations. These factors are predominantly presented in a descriptive and case-by-case manner, which does not provide insight into their relative significance. The identified factors were classified into three main categories: 1. Contextual factors; these lie outside the domain of the research process; 2. Quality of the evaluation research; and 3. Interaction factors, which are situated. The latter two categories lie within the domain of the research process. The three main categories include several subcategories, which are described successively below (Table 2).

### 1 Context

The first category of factors that can influence the impact of ex-post legislative evaluations is the context in which these evaluations are commissioned, conducted and landed. These contextual factors may determine the need and necessity for an ex-post legislative evaluation, as well as the attainability of impact after the evaluation has been conducted. In other words, the use of evaluation results may depend on various contextual factors. In this scoping review, we see the concept of context as a factor that is outside the evaluation process but with which the evaluation is concerned; it is the setting in which the evaluation process takes place.

The first contextual factor, as found in this study, relates to the type of legislation being evaluated and the way in which ex-post legislative evaluations are initiated. Early literature notes that not every law is suitable for evaluation research (Gevers, 1995; Legemaate, 1997; Veerman, 1991). Laws, for example, can be codifying in nature, be complex (Gevers, 1995; Legemaate, 1997) or vague in content, and have different or contradictory objectives that are not always expressed (Veerman, 1991). This complicates the evaluation and hinders the drawing of conclusions, resulting in little benefit from the evaluation. Legislation with a modifying character, on the other hand, introduces new processes or practices in society and is more verifiable (Gevers, 1995). This type of legislation works more as a policy tool and its evaluation can be valuable to the legislative process (Gevers, 1995). In this regard, early case studies have shown that the degree of elaboration of norms in a law or regulation affects the likelihood of using the evaluation results (Winter et al., 1990). If the norms of the statutory regulation are less developed, evaluations that require the presence of a legal perspective in the legislative evaluation are more likely to be used (Winter et al., 1990).

Which law is evaluated may depend on how an ex-post legislative evaluation is initiated: based on an evaluation clause in the law itself or at the request of, say, the political domain (Bussmann, 2010). The way an evaluation is initiated appears to influence the impact the evaluation subsequently has. A pronounced situation is seen in the European Union (hereafter: EU) context where evaluations are conducted *ad hoc* at the request of the European Parliament. These evaluation requests are presumably based on a strategic goal, giving the ex-post legislative evaluation a strategic function. An example was mentioned in one of the included studies. The results indicated that ‘the Commission prioritises evaluating legislation for which the chances of non-compliance are relatively high, and that evaluation may at least partly be initiated to scrutinise member state implementation’. (p. 653) (Van Voorst & Mastenbroek, 2017).

This is different from ex-post legislative evaluations that are conducted systematically on the basis of an evaluation clause included in the legislation itself (Bussmann, 2010). The main reason for conducting such legislative evaluations is the legal obligation to do so, as shown by two comprehensive studies on the European Commission (Mastenbroek et al., 2016; Van Voorst, 2018). They are conducted because it is mandatory, not because there is a specific interest in doing so. Those mandatory evaluations may be seen as less politically relevant (Klein-Haarhuis & Parapuf, 2016) but do ensure that the specific law is put back on the political agenda (Winter, 1996). There seems to be a perception that mandatory systematic ex-post legislative evaluations have less impact due to less current relevance. This is supported by data from an empirical study which showed that the Commission’s compliance with such clauses only occurred in about half of the cases (Van Voorst & Mastenbroek, 2017).

The second contextual factors identified in the literature is the importance of the openness to evaluation results and the willingness to implement the evaluation results, as mentioned by several authors (Doust & Hastings, 2019; Legemaate, 1997; Van Voorst & Zwaan, 2019; Veerman, 1991). In the first place, the legislative process must allow for the results of an ex-post legislative evaluation to have impact. The way the process is designed may influence the extent to which results will be used. In order to feed evaluation results into the legislative process, there must be a place in this process to adapt legislation based on an evaluation (Bussmann, 2010). A fixed routine can create a learning system, also known as a ‘regulatory cycle’ (Klein-Haarhuis & Parapuf, 2016). In the Netherlands, a policy response should be formulated within three months of the delivery of the evaluation report. This response should report what the minister intends to do with the study (Klein-Haarhuis & Parapuf, 2016). It is ultimately up to the various actors to incorporate the evaluation results into the legislative process. In particular, one of the case studies showed that if parliament has no interest in the evaluation report, little is done with the recommendations given in that report (Doust & Hastings, 2019). Although evaluations can never oblige the legislator to amend the law, it is important that there is a willingness to tackle actual bottlenecks by amending the law if necessary or by incorporating the evaluation results into policy (Klein-Haarhuis & Parapuf, 2016; Legemaate, 1997). Otherwise, evaluation can be meaningless, because without the effective support of the parliament and the government, an ex-post legislative evaluation can never be properly implemented in the decision-making process (Hendriks, 2000). A robust relationship between the legislative and executive branches seems to be necessary for this (Van Aeken, 2011).

An important reason for acting or not acting on the evaluation results lies in the function given by stakeholders to the ex-post legislative evaluation. Several functions can be assigned to ex-post legislative evaluations, which can influence the way they are used. An evaluation can, for example, be focused on efficiency, but also on process optimisation or the investigation of side effects. The literature suggests that different stakeholders assign different functions to (the same) legislative evaluation (Veerman, 1991), such as a tactical, symbolic or legitimising function (Nelen, 2000). Depending on these functions, the evaluation results will be used to a greater or lesser extent (Eijlander, 1993; Veerman, 1991). If the function, for example, is given to gain knowledge about the efficacy of the law in practice, it is more likely to be acted upon than if the function is procrastination (Veerman, 1991). Another example is described in a case study on the Dutch Director Liability Act where the House of Representatives had doubts about the usefulness of this act and its burden on business. The Minister of Justice proposed an evaluation in order to ensure a majority for the bill, indicating a tactical function. For the members of parliament, the acquisition of knowledge was the primary function (Veerman, 1991). Different interests give different functions to legislative evaluations that determine how they are used. Another example takes place in the UK, where both Houses have different motivations and approaches to oversight, resulting in sporadic or limited post-legislative scrutiny. The nature of the incentive is crucial to explain the extent of such scrutiny (De Vrieze & Norton, 2020).

The final contextual factors relate to the social and political atmosphere in which ex-post legislative evaluations are conducted. As the literature shows, the political and administrative decision-making context is considered an important factor influencing the use of evaluation research (Klein-Haarhuis & Parapuf, 2016; Van Voorst & Zwaan, 2019). This is supported by two systematic studies on the use of ex-post legislative evaluations in both the Netherlands (Winter et al., 1990) and the EU (Zwaan et al., 2016) showing that the level of political conflict is the most important variable to explain differences in the use of evaluation results. The perception from the literature is twofold, on the one hand it is mentioned that controversial evaluations during the legislative process are most likely to be used (Van Voorst, 2018; Zwaan et al., 2016). On the other hand, there is a perception in the literature that when political conflict is high the impact of using evaluation results is limited (Bussmann, 2010; Eberli, 2018). Unforeseen political issues can also arise and affect the political process and the use of evaluation findings (Bussmann, 2010).

Regarding the social sphere, the literature argues that the level of involvement of so-called interest groups can influence the impact of ex-post legislative evaluations. For example, one study concluded that ‘Such groups may have no formal veto over policy proposals, but they can put pressure on policy-makers to ignore or implement evaluation results, either directly via lobbying or indirectly via the media. To produce a policy that satisfies a wide range of actors, policy-makers may prioritise such interest group preferences over evidence from evaluations’ (pp. 368–369) (Van Voorst & Zwaan, 2019). On the other hand, policy-makers can be stimulated to include evaluation results in the policy by strong public opinion on the matter, for example in the media (Van Voorst & Zwaan, 2019).

#### 2 Research quality

The second category of factors influencing the impact of ex-post legislative evaluations is associated with the research quality itself and the group of researchers conducting the evaluation. First, literature highlights the importance of an independent position for those conducting the evaluation, particularly for sensitive or politically charged topics (Vranken & Van Gestel, 2008; Winter, 1997). Differences exist between countries, as ex-post legislative evaluations can be conducted by various institutions with varying levels of independence from parliament based on the parliamentary culture. For instance, in the United Kingdom, post-legislative scrutiny can be performed by parliamentary committees, commissions, external working bodies, or independent state agencies, depending on the nature of incentive: formalistic review is suitable for officials, while evaluative oversight is better undertaken by legislators (De Vrieze & Norton, 2020). In the Dutch context, ex-post legislative evaluations are typically conducted by external parties like research institutes or university departments, where the British subdivision is not made. The literature often suggests that external execution can enhance research independence (Hendriks, 2000; Van Aeken, 2011; Vranken & Van Gestel, 2008), allowing researchers to maintain a greater distance from the law and policy (Hendriks, 2000). Government departments or parliamentary committees may be too closely involved with existing regulations (Van Humbeeck, 2000b), and executive-led research tends to lack independence in the literature (Doust & Hastings, 2019).

However, opinions in the literature differ about the degree of independence. On the one hand it is argued that the lack of independence can have an obfuscating effect because reliability and validity can be doubted (Vranken & Van Gestel, 2008). On the other hand, it is argued that too much independence creates a separation between the ex-post legislative evaluation and policy development, which can also reduce its credibility (van Humbeeck, 2000a). This can lead to evaluations of legislation not being taken seriously and therefore not being used. Since the credibility of the report is also an important factor in the use of the ex-post legislative evaluation, it is argued that there should be a proper balance between the level of independence and sufficient involvement of the commissioning party (Vanlandingham, 2011). Otherwise, if the evaluators are too concerned with the incentives and objectives of the regulatory bodies on which their administration depends, ‘evaluation research degenerates toward a formalistic ritual without real content or impact’ (p. 63) (Van Aeken, 2011). Early research comparing five case studies on the evaluation of Dutch laws found that when a balance is achieved between policy proximity and independence, the likelihood of using evaluation results is higher (Winter et al., 1990).

Several authors consider the methodological quality of the evaluation research essential for the use of evaluation results (European Court of Auditors, 2018; Hendriks, 2000; Winter, 1997). In The Netherlands, quality is stimulated by appointing a guidance committee, among other things (Klein-Haarhuis & Parapuf, 2016). This committee monitors the progress of the evaluation study and meets several times during the evaluation process, usually to discuss successively at least the research design, progress and the draft and final versions of the report. They can make timely interventions and adjustments if the research is not going according to plan (Klein-Haarhuis & Parapuf, 2016).

The way in which ex-post legislative evaluations are generally conducted involves a combination of legal and empirical research. According to early researchers, the legal part in particular increased the use of the evaluation results (Winter et al., 1990). Other authors emphasise the standardisation of research methods, which, despite being able to constrain the flexibility of using research methods, proves important for the effectiveness of evaluations (Van Humbeeck, 2000b). In contrast, the literature also argues that methodological quality is less decisive for the use of evaluation results. For example, a case study on the comparison of the Dutch and Danish evaluation on the promotion of joint parenthood after divorce showed that a good evaluation does not necessarily lead to increased use of evaluation results (Jeppesen de Boer, 2014). The Danish evaluation was very detailed and comprehensive, but the results of the ex-post legislative evaluation were hardly taken into account in the subsequent amendment of the law. Early case studies also showed that research quality does not always determine the extent to which evaluation results are used. Despite low quality, evaluations did contribute to influencing views (Winter et al., 1990). The authors note that if there is consensus, there is not much need for hard research findings (Winter et al., 1990).

The actual findings of an ex-post legislative evaluation can also determine the use of the results. An included meta-analysis of ex-post legislative evaluations showed, for example, that the use of evaluation results was promoted if the results confirmed the usefulness of the deployed policies and related legislation (Veerman et al., 2013). Another systematic research study showed that recommended amendments can have a significant effect on the use of evaluation results. The study showed that the chances of an evaluation being used increases by 2.1% for every extra amendment proposed in the evaluation report (Zwaan et al., 2016). The strength of the recommendations and the action they call for also seem to have a strong influence on the acceptance of the recommendations in the research report (Caygill, 2019). Therefore researchers tend to focus on recommending small and medium actions to increase the likelihood of acceptance, as suggested by a systematic research study on post-legislative scrutiny recommendations in the UK parliament (Caygill, 2019). This was also shown by a multiple case study in which the authors concluded that recommendations that do not deviate, or only a little, from the existing legal system are more likely to be implemented (Winter et al., 1990).

#### 3 Interaction

The third category of factors that influence the impact of ex-post legislative evaluations is the interaction between evaluation researchers and stake-holders that takes place both before the evaluation assignment as well as during and after the execution of the ex-post legislative evaluation.

First, the interaction between the commissioner of an ex-post legislative evaluation and the research group begins with the commissioner’s assignment. Thus, a clear research question is important because this question can influence the entire research process, which in turn can influence its impact (Veerman, 1991). Veerman (2014) suggests that a non-specific but global evaluation assignment may lead to disappointment afterwards. In such situation, it often only becomes clear afterwards what information the commissioner would like to have investigated. In that case, the results are used less and perhaps only received as knowledge, which could have been avoided with a more focused research assignment at the beginning of the evaluation process (Veerman, 2014). Case studies showed the importance of properly translating the policy question into the research question to maximise policy relevance (Winter et al., 1990). From the perspective of achieving impact, it is therefore important that policy and legislation on the one hand and researchers on the other consult on what is most relevant to investigate. It also means that during the research process, the commissioner should keep in touch with the researchers about the status of the research (Veerman, 2014). The authors of a systematic research article even claimed that contact and consultation between the commissioner and researchers during the evaluation process is a crucial way to increase the likelihood of use (Klein-Haarhuis & Parapuf, 2016).

Several authors point out the importance of interaction during the research process, not only with respect to the commissioner, but also the legislative stakeholders. The literature first notes that this can be done by addressing the recommendations in the evaluation report not only to the legislator, but also to the courts, the administration (Scheltema, 2002) and society. However, this is only done at the end of the evaluation when the research is already completed. Several authors conclude that involving different stakeholders at an earlier stage of the evaluation process leads to greater impact. For example, as shown by a case study, good contact with policy makers during the evaluation process led to policy relevance, which subsequently led to reasonable use of evaluation results (Winter et al., 1990). Vanlandingham (2011) also showed that researchers who were able to consult regularly with stakeholders had more value and impact in the legislative process. Another example was given in a case study, where the authors emphasised society involvement and even advocated a more bottom-up approach by directly engaging the people at all stages of the review process to improve public engagement (Moulds & Khoo, 2020).

A more or less ‘last’ step in the interaction between researcher and stake-holders in the broad sense, is the way in which evaluations results are shared. Although earlier research expected that the manner of publication may influence the use of ex-post legislative evaluations (Veerman, 1991), systematic research on ex-post legislative evaluation offices’ efforts to promote utilisation concluded that ‘report distribution activities were not found to be statistically related to utilisation differences’ (p. 90) (Vanlandingham, 2011). However, the lack of use of the evaluation results is sometimes explained by the way in which the information is presented (Poptcheva, 2013) and the fact that the information is not available (Van Schagen, 2020). It is important that evaluation results are available and usable. The researchers play an important role here because they can influence the way the evaluation report is disseminated, for example by giving presentations or writing a scientific article summarising or reflecting on the evaluation results (Klein-Haarhuis & Parapuf, 2016). Additionally, the language in which the report is written may affect its use. ‘Evaluations are usually only published in one language (generally English), which hinders their usability for stakeholders and citizens’ (p. 4) (Poptcheva, 2013).

Last, the reviewed literature shows timing as an important part of interaction. Some of the studies claim that timing is important into the extent to which the evaluation results are used and thus the potential impact they can have. Several authors stated that evaluation results are often not available when key decisions must be made (Vanlandingham, 2011; Vranken & Van Gestel, 2008): an evaluation process may come too late to lead to further processing (Klein-Haarhuis & Parapuf, 2016). Also the European level, the timely availability of ex-post legislative evaluations is also crucial in allowing the results to be used in (exante) impact assessments, and this shows the importance of strictly enforcing the Commission’s ‘evaluate first’ principle (Van Voorst, 2018). In order to maintain the regulatory cycle, it is crucial that the ex-post legislative evaluation is available before the (ex-ante) impact assessment is conducted (Golen & Voorst, 2016).

On the other hand, evaluations can also be conducted too early and thus have a minimal impact. Legislators are often impatient and want an early evaluation for various reasons. However, this means that these evaluations are conducted at a time when the policy area to be examined is still in full swing. If the ex-post legislative evaluation is conducted in a very ‘early’ stage, the picture of the results may not yet be complete (Vranken & Van Gestel, 2008) or it could be too early for adequate conclusions to be drawn (Klein-Haarhuis & Parapuf, 2016). The pitfall, therefore, is that the evaluation will provide misleading information and cannot lead to quality improvement (Winter, 1997).

## Discussion and conclusion

The importance of ex-post legislative evaluations is recognised worldwide. Countries conduct ex-post legislative evaluations to varying degrees to assess effectiveness in practice and to improve laws and regulations. These evaluations are meant to be used. It is therefore important they are not only conducted but actually acted upon. Otherwise, an ex-post legislative evaluation will be relegated to a formal ritual with little or no effect and whose use-fulness can be questioned. This scoping review clarifies the factors that influence the impact of these ex-post legislative evaluations.

One of the most comprehensive studies referred to in this scoping review is the study by Winter et. al. (Winter et al., 1990) in which a framework was established based on empirical research. Using five case studies and a survey of 25 legislative evaluations, the authors systematically examined, among other things, the determinants of the use of evaluation results. They examined the characteristics of the regulation (e.g. determinacy, vagueness and intrusiveness), characteristics of the evaluation process (e.g. policy proximity, independence and elaborate standardisation), characteristics of the evaluation product (e.g. research quality, policy relevance and feasibility of recommendations) and the degree of conflict in the decision-making context. The study found that the determinacy and vagueness of the legal regulation was not found to be a clear driver of the use of evaluation results. However, the intrusiveness of the regulation does play a role in the likelihood of use. Highly intrusive regulation has a closed context in which room for making changes is limited.

The study showed with regard to the evaluation process, that ex-post legislative evaluations are more likely to be used when there is a proper balance between policy proximity and independence. In addition, it was found that there is a higher probability of usage when the norms in the legal regulation are less elaborated. Therefore, the impact of the ex-post legislative evaluation will be directed at refining and elaborating upon de underdeveloped norm.

With regard to the evaluation product, it was found that evaluation results are used especially if they are policy-relevant. This also transcends low research quality: even if an evaluation is of low quality, the evaluation can be used to a great extent if the policy relevance is present. With regard to policy relevance, it was concluded that this is greater when the policy question is properly translated into the problem definition of the study, when there is periodic consultation with the client and the evaluation report is published at the right time (Winter et al., 1990). The likelihood of use is, however, higher if the recommendations differ slightly from the existing situation (Winter et al., 1990).

This scoping review, based on more recent and international publications, confirms the importance of characteristics of the evaluation process (e.g. policy proximity and independence) and characteristics of the evaluation product (policy relevance and feasibility of recommendations), but displays more factors influencing impact of ex-post legislative evaluations.

In contrast to the study of Winter et al. (1990), where the categories to be analysed were predetermined, we maintained an open-minded approach in this scoping review and established a categorisation based on the existing literature on the factors that (may) affect the impact of ex-post legislative evaluations.

Caution is needed as not all studies are based on robust empirical research. Over the years, empirical studies have had a limited presence in investigating the factors that influence the impact of ex-post legislative evaluations. However, despite this limitation, the included empirical studies have identified additional factors such as the evaluation function (Nelen, 2000), evaluation initiation (European Court of Auditors, 2018; Mastenbroek et al., 2016; Van Voorst, 2018; Van Voorst & Mastenbroek, 2017), timing (Golen & Voorst, 2016; Klein-Haarhuis & Parapuf, 2016; Van Voorst, 2018) and the interaction between researchers and those involved (Klein-Haarhuis & Parapuf, 2016; Vanlandingham, 2011). In addition, this scoping review also included case studies and opinion articles that noted or suggested additional factors. The results point to a clear tripartite division of factors that matter to the degree of impact of ex-post legislative evaluations.

### The relevance of context for impact

First, the literature shows that the context of the evaluation process plays an important role in the impact of ex-post evaluation of legislation. Context is determined by the initiation and function of the evaluation, stakeholders’ openness towards the evaluation results and the political and social context.

A recurring observation is that the impact of ex-post legislative evaluations is influenced by the willingness of actors, such as the commissioner and policy-makers, to adopt the evaluation results and have them translated into legislation and policy. Such willingness also depends on the extent to which actors could use an evaluation for their specific interests and agenda (e.g. legitimisation, monitoring or political-strategic purposes). For a given ex-post legislative evaluation, the interests of each actor may vary, owing to their distinct concerns and objectives. In particular, political interests seem to play a role in deciding whether or not to follow up on ex-post legislative evaluations. These interests may already come into play at the commissioning stage of an ex-post legislative evaluation. Such an evaluation may be actively requested or be the result of a mere legal obligation. An ex-post legislative evaluation that is actively requested is likely to have more impact, as at least one actor has a specific interest in the results of the evaluation. This could, for example, be a political interest. However, the influence a political interest has on the use of evaluation results is ambiguous. Some authors argue that political involvement ensures that the impact of ex-post legislative evaluations is more present, while others conclude that significant political attention ensures that, on the contrary, evaluation results are not used.

### The relevance of research quality for impact

Second, the literature shows that some factors related to the research quality may affect the impact of ex-post evaluations of legislation. This includes both the quality of the research (methods used, research findings and recommendations) and the research group (independence of the research group). The results show that, with regards to recommendations made in the evaluation report, the use of evaluation results was promoted if the evaluation report made recommendations or if the evaluation results confirmed the usefulness of the implemented policy and related legislation. Surprisingly, little evidence was reported on the effect of the methodological quality as determining factor for the impact of an ex-post legislative evaluation. One aspect of research quality, the independence of the research, is frequently discussed in the literature. Independent research is described as an important condition for the reliability and validity of evaluation research, especially if the topic is sensitive or politically charged. Independent research contributes to the objectivity of research findings and can be achieved, for example, by choosing an external research group. However, too much independence is not considered desirable. Evaluators aim to speak the truth to powerful entities instead of lobbying for specific interests. However, if the focus on independence becomes too extreme, it can lead to a lack of effective communication and the truth not being conveyed to anyone (Vanlandingham, 2011).

### The relevance of interaction for impact

The third and last factor that influence the impact of ex-post legislative evaluations, as described in the literature is the interaction between the researchers on the one hand and the recipients of the evaluation results on the other. Recipients of evaluation results may include both the commissioner of the evaluation, the political domain, the legal domain and society as a whole. The literature shows that during the whole evaluation process, interaction with these different actors plays an important role in the possible impact of ex-post legislative evaluations. Interaction already starts with the commissioning of an evaluation assignment. During the process, interaction with stakeholders such as the commissioner and participants is also reported to be a crucial way to increase the likelihood of use. Some scope for client involvement helps to create support after delivery of the evaluation report. There are also opportunities to increase impact at the end of the evaluation process by the way in which evaluation results are available and presented. For the whole process, the timing of the evaluation plays a crucial role. In order to gain as much impact as possible, evaluation results should be available when key decisions must be made.

### Research findings in the light of Alkin and King’s study

The three categories outlined in this scoping review are broadly consistent with those described in Alkin and King’s (2017) study, with both studies highlighting the significance of recognisability and relevance to end-users in stimulating evaluation use. The research quality and interaction factors we have describe align with those presented by Alkin and King (2017), but there are significant differences in their context. These may be attributed to the researcher’s perspective in our analysis, as well as the unique characteristics and stakeholders of the legislative domain as compared to the broader field of policy and programme evaluation. Unlike policy and programme evaluations, ex-post legislative evaluations primarily impact the design of legislation and thus the impact is mainly found in the legal domain. Specific mechanisms, such as elaborating norms in legislation based on evaluation results, are unique and not found in the broader evaluation literature. Another notable difference is the emphasis in the legal literature on the relevance of the initiative to commission the evaluation.

On the other hand, the ex-post legislative evaluation field may benefit from the more established ideas in the broader evaluation literature based on more extensive and empirical research. This provides a call for the expost legislative evaluation field to leverage the broader evaluation literature and conduct more empirical research. Additionally, the field of expost legislative evaluations could contribute to the field of policy and programme evaluation by providing insight in how evaluation results are integrated into the legal domain.

### A current dilemma

The results of this scoping review also reveal a dilemma for researchers: how to combine productive interactions with the stakeholders of an evaluation while ensuring the independence of the evaluation. Conducting an evaluation and thereby having impact requires the convergence of two worlds, the evaluator on the one hand and the recipient or user of the evaluation results on the other.

The context of an evaluation is a given that researchers have to deal with when conducting an ex-post legislative evaluation. Researchers cannot control the context and have to ‘work’ with it, but researchers can take the contextual factors into account. They can seek to interact with the various stake-holders in this context and respond to the given context. This interaction can take place at different stages of the evaluation process (e.g. in the preparation phase, the execution phase or the implementation phase). Interaction works in two ways: interaction allows researchers to respond to the field to increase the likelihood of impact of the ex-post legislative evaluation. On the other hand, interaction may influence researchers during the evaluation process, making evaluation results more responsive to questions in the field. The right balance is needed, which has also been described by Winter et al. (1990). While research independence is considered essential for impact, researchers depend largely on the input from respondents during the evaluation process, which can reflect the strategic agendas of involved parties. Balancing impact and independence requires understanding these agendas, aiding researcher in presenting results that resonate with these parties. However, evaluation results and conclusions should not be the result of stakeholder pressure; they should be based on good research quality. On the other hand, evaluation conclusions will have more impact when they align with the strategic agendas of involved parties. This is a dilemma that places demands on the evaluation process, such as in the form of an external guidance committee. This dilemma also warrants more in-depth research, that will be relevant for the specific field of ex-post legislative evaluations and the broader field of policy and programme evaluation.

Another aspect warranting in-depth investigation is the complexity of responsible institutions for ex-post legislative evaluations across countries, combined with diverse conceptualisations within parliamentary cultures. These variations in approach arise from distinct contextual backgrounds and frameworks in each nation, resulting in diverse methods – like the UK’s customised approach and the Netherlands’ reliance on external entities. This scoping review underscores how the significance and positioning of evaluations affect their outcomes, implying potential for enhanced comparative research despite its absence in this study.

## Figures and Tables

**Table 1:** Final scientific search strategies.

Web of Science, Worldcat Discovery and Legal Intelligence
***Dutch search terms***
*1st strategy*
TI = (wetsevaluatie* OR “Evaluatie wet*” OR “evaluatie regel*”)
*2nd strategy*
TI = (wetsevaluatie* OR “Evaluatie wet*” OR “evaluatie regel*” AND aanpak OR uitvoering OR method*)
*3rd strategy*
TI = ((wetsevaluatie* OR evaluatie wet* OR evaluatie regel*) AND (impact OR gevolg OR invloed OR effect**))
***English search terms***
*1st strategy*
TI = (’Legislative evaluation* OR “Law evaluation*” OR “Evaluation of legislation*” OR “Legal evaluation*”)
*2nd strategy*
TI = ((legislative evaluation* OR law evaluation* OR evaluation of legislation OR legal evaluation*) AND (method*))
*3rd strategy*
TI = ((legislative evaluation* OR law evaluation* OR evaluation of legislation OR legal evaluation*) AND (impact OR influence OR result* OR utilization))

**Table 2:** Factors influencing the impact of ex-post legislative evaluations.

	Factors influencing the impact of ex-post legislative evaluations
1	**Context** − Evaluation initiation and function − Political and societal influence − Openness to the evaluation results
2	**Research quality** − Composition and independency of the research group − Methods used − Quality and content of evaluation report
3	**Interaction** − Interaction between researchers and client or participants − Presentation and availability of research results − Timing
